# Regulatory Aspects of Pharmaceuticals’ Exports in Gulf Cooperation Council Countries

**DOI:** 10.4103/0975-1483.80305

**Published:** 2011

**Authors:** S Pateriya, MD Janodia, PB Deshpande, VS Ligade, KB Talole, T Kulshrestha, Y Kamariya, PB Musmade, N Udupa

**Affiliations:** *Department of Advanced Pharmaceutical Studies, Manipal University, Manipal-576104, India*; 1 *Manipal College of Pharmaceutical Sciences, Manipal University, Manipal-576104, India*; 2 *Zydus Research Center, Ahmedabad, India*

**Keywords:** Exports, Gulf cooperation council, pharmaceutical, regulatory

## Abstract

The Gulf cooperation council (GCC) region is considered as “Emerging market” for pharmaceutical export and bilateral trade. The understanding of the regulatory requirements of this region can be beneficial for pharmaceutical export. Some incidents of the year 2008-09, like recession or economic slowdown in highly well-off and regulated market of the EU and US, raised the demand for alternate destinations for business. The regulations of Gulf countries are encouraging the import of quality generic products, which can be good news to the Indian drug manufacturers.

## INTRODUCTION

### Indian Pharmaceutical export: present scenario

**Figure 1:**
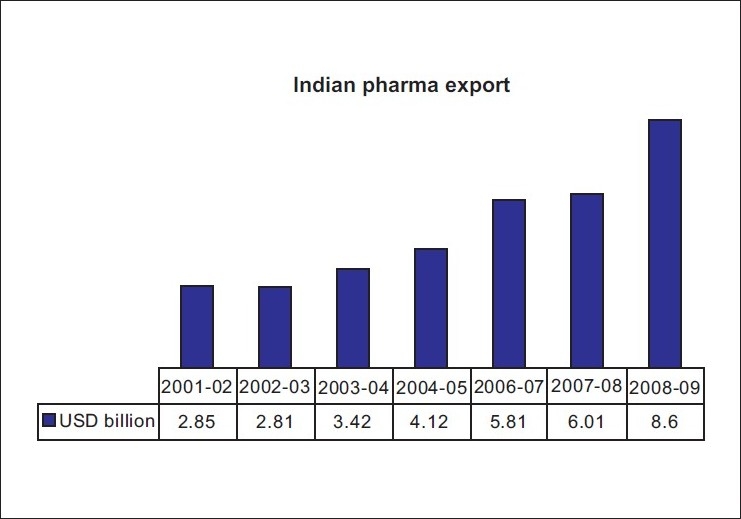
Statistics on Indian pharmaceutical exports [Source: Singh S, Drugs Regulatory Reforms & Policy Initiatives- India accessed http://www.assocham.org/7km2009/presentations/Day_2_27_11_09/Surinder_Singh_DCGI.pdf]

The Indian pharmaceutical industry, which is one of the major manufacturers of multi-source generic drugs, has a broad spectrum of export of pharmaceuticals all over the world. The exports of drugs, pharmaceuticals, and fine chemicals for the year 2008-09 stood at US $ 8.6 billion, registering a growth of about 29% over the last year.[1,2] This is shown in Figure 1.

The exports of drugs, pharmaceuticals and fine chemicals from India have grown at a compounded annual growth rate (CAGR) of 17.8% during the five-year period 2003-04 to 2007-08.[1] The share of pharma exports to total exports is 2% over the last 12 years.[3]

### Need of the hour

Traditionally, larger pharmaceutical markets like the US, Europe have been the prime target markets for Indian pharma companies. But since the last few years, there has been a remarkable depression in the export profile of India to these countries. The export of pharmaceuticals to the US has drastically declined at the rate of 36.8%.[4]

At the same time the other destinations of export are not only yielding the same ratio of profit but are also increasing at an appreciable rate. Many new windows for trade are opening for India. For example, export to African countries has increased from 10-15% in the year 2007-08.[5]

Therefore, it is time to look at other segments of the market which is by and large called ‘emerging market’ and can increase our export rate including our traditional customers.

## GULF COOPERATION COUNCIL[6]

### Export opportunities for India:[7]

Based on responses from 215 representatives of leading pharmaceutical manufacturers in India and those registered in the six GCC countries, the FICCI study found an overwhelming keenness to tap the GCC market.

According to a FICCI survey report “There is a considerable scope for increasing our exports of drugs and pharmaceutical products to GCC countries, namely Saudi Arabia, Kuwait, Bahrain, Qatar, United Arab Emirates and Sultanate of Oman [Tables 1 and 2].”

**Table 1:** General information about the Gulf Cooperation Council countries

Member states	Saudi Arabia Kuwait Oman Bahrain Qatar United Arab Emirates
Demographics	Total population estimated at 45 million inhabitants The GCC is a cooperation organization having different domains including health The various councils of ministers of the participating countries meet twice a year to discuss existing and new cooperation issues

**Table 2:** Opportunities in the Gulf Cooperation Council market

GCC countries- 80% demand of pharmaceuticals through import Indian export to GCC countries- 119.25 USD billion (2002-03) Largest trading partner after US - 6.8 USD billion business annually[7] Annual increase of population is more than one million - Great potential for healthcare sector Economic development boards (EDB), joint ventures and economic liberalization by GCC countries- Opportunities for Indian investors[19,20] Less custom duty on pharmaceuticals- duty-free (Saudi Arabia & UAE) and 4-5% by all the remaining GCC members.[18]

### New juncture

#### Gulf Cooperation Council regulatory authorities

Gulf Central Committee for Drug Registrations (GCC-DR)

- Approved in May 1999.
- Located in the executive office for Health Ministers, Riyadh, Saudi Arabia.

**Drug registration**: there are two processes of drug registration;

- Centralized registration procedure
- Decentralized registration procedure

### A. Centralized registration procedure:[21]

**Figure 2:**
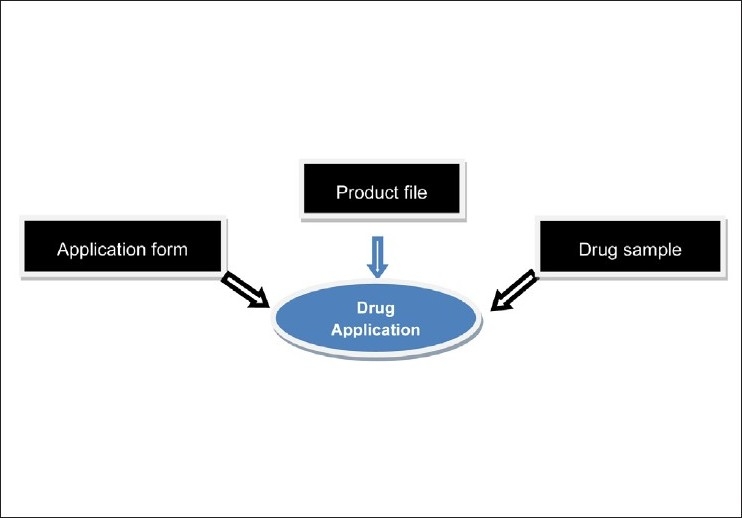
Components of dossier

1. The executive office of GCC-DR assumes the receipt of registration files after ensuring the fulfillment of registration requirements and upon duly filling the following forms:
  - The drug companies’ registration form.
  - A pharmaceutical chemical entity/ preparation registration form.
2. Eight complete files for each chemical entity and 17 samples have to be submitted to the executive office and two samples shall be dispatched to each country along with registration file.
3. Every country shall study the registration files forwarded to it and then return those files with its recommendation to the committee. The procedure is shown in Figure 2.
4. The company needs to provide the laboratory for the analysis of standard materials, methods etc.
5. The executive office dispatches the samples of chemical entity to reference-accredited laboratory for the analysis.
6. After approving the registration of company andor chemical entity centrally, the remaining authentication and documentation, fees are finalized on country basis, as per their prescribed and established policies. The fees for centralized procedure is shown in Table 3.
7. The executive office issues the registration certificate.
8. The companies reserve their rights to lodge their grievances to the executive office within a period of two months effective from the date of notification about the registration by GCC-DR.

**Table 3:** Fees for drug registration

Company registration		Product registration	
SR. 5000 which shall be the 50% fee against studying the company’s file	SR. 5000 which shall represent 50% of remainder fee upon the final consent and approval for their registration	SR.3000 which shall be 50% of registration fee against studying the company’s file	SR. 3000 which shall represent 50% of remainder fee upon the final consent and approval for their registration

### Fees

#### The validity of the central registration

- The central Gulf committee’s resolutions for drug registration are binding for the consolidated purchasing.
- All countries must sanction and approve the export price, which has been approved by the committee upon completion of the registration procedures in the country.

### Issue of Centralization of registration of drugs

It is not mandatory to centralize the registration of drugs in GCC, as of now. But for special classes of drugs, registration through the centralized process is necessary. These are as follows:

1. Generic drugs for which bioequivalence studies cannot be done, e.g. inhalable medicines and some nasal inhalers.
2. Drugs supported by biotechnology for which bioequivalence studies cannot be done and which require clinical or pharmacodynamic studies.
3. Drugs with narrow therapeutic spectrum, which are administered orally.

### B. The Decentralized registration procedure:[8]

#### Registration regulations in major countries of GCC

Although there is a centralized and quite harmonized process for drug registration in GCC countries, the regulatory requirements of a few big countries like Saudi Arabia and UAE are separate. These countries have their well-established regulatory system and its enforcement.

In this study, we will discuss briefly the registration requirements of multi-source generic products of the following GCC countries:

1. Saudi Arabia
2. Bahrain
3. Kuwait
4. UAE

## DRUG REGISTRATION REGULATIONS OF THE KINGDOM OF SAUDI ARABIA[8–10]

The Saudi Food and Drug Authority [SFDA] is the main drug regulatory body of Saudi Arabia. SFDA prefers the drug dossier submission in electronic format (eCTD). The SFDA has approved more than 6177 drugs of different strength and formulations, till May 20, 2010.[11] Drug registration requirements and procedure is as follows:

### Drug Registration

#### Online filing of application

The appropriate application form can easily be downloaded from the official website of SFDA i.e. http://www.sfda.gov.sa/En/Drug.

Once the application is submitted, a reference number is given to the applicant to facilitate communication with SFDA.

The applicant is needed to make an appointment with the SFDA office to hand over the application. The earliest appointment can be scheduled 1 to 12 weeks in advance. The applicant can reschedule a week before the appointment. An automatic reminder will be sent 3 days before the appointment. Target performance timeline for SFDA is shown in Table 4.

**Table 4:** Target performance timelines[10]

Process	Target performance timeline (days)
Marketing authorization (MA) application for generic drugs	165
Marketing authorization (MA) application for new chemical entity (NCEs)	290
Marketing authorization (MA) application for biologics	290

### Registration process:


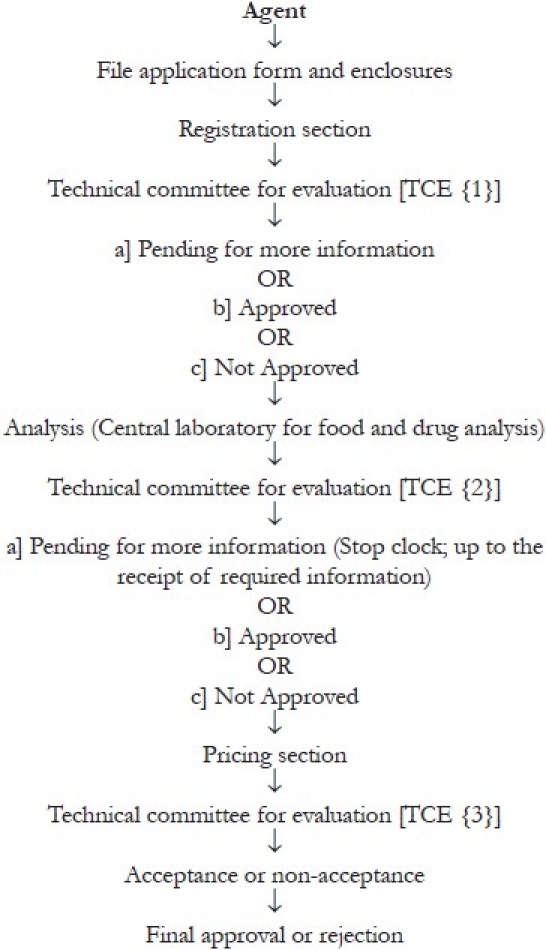


### Stability data requirements[12]

SFDA recommends the GCC guidelines for stability study data preparation. GCC countries come under climatic Zone III and IVa (Hot and dry and hot and humid). Three primary batches are recommended by the GCC guidelines. For generic drug product long-term stability study supporting the complete proposed shelf-life should be submitted.

## KINGDOM OF BAHRAIN-DRUG REGISTRATION REQUIREMENTS[13]

The regulations for pharmaceutical registration in Bahrain are almost similar to the other GCC countries. All types of necessary information is required by the Ministry of Health, Bahrain but as compared to the other GCC countries, they also focus on the details of the company profile and several types of business activities like current business merger by the company, etc. The following table provides the basic information required for the registration of a drug in the Kingdom of Bahrain and other GCC countries.

## KUWAIT: DRUG REGISTRATION REQUIREMENTS

Medicines in Kuwait are regulated for quality, safety and efficacy standards, price control, and patent protection. Kuwait has 40 years of experience of a regulatory system and plays a prominent role in the GCC. The Kuwait food and drug authority (KuFDA) is the head regulatory agency, which follows the ministerial decree 302/80 to register pharmaceutical products.

According to Dr. Reem Al-Essa, Head of the Licensing Division of the Kuwait Drug and Food Control, “Kuwait is facing an overwhelming regulatory challenge reflecting the rapid advancement of the regulatory services with limited resources possibly influencing patients’ timely access to medicines.”[14] The Pharmaceutical manufacturing environment is not very competitive in Kuwait because of the very small population and the lack of the proper expertise to establish a good base of manufacturing practices for the pharmaceutical products within the country. There is only one manufacturing company (Kuwait-Saudi Pharmaceutical Industry Co- KSPICO) in Kuwait. This company produces generic medicines.

### Enclosures required for drug registration in GCC countries[8,9]

The documents required to be submitted to fulfill different regulatory requirements for drug registration in GCC countries are summarized and compared in Table 5 and 6. Five countries have been taken for the comparison. They are Saudi Arabia, UAE, Oman, Bahrain, and Kuwait.

**Table 5:** Drug registration requirements in Saudi Arabia, Bahrain, Kuwait and UAE

Saudi Arabia[8–10]		Bahrain[13]		Kuwait[15,16]		UAE[17]	
Format	CTD format. eCTD recommended. Module 2 to 5: according to ICH CTD format. Module 1: regional requirements: Cover letter Table of contents (ToC) Application form Product information: summary of product characteristics (SmPC), product information leaflet (PIL) and labeling – all in WHO template format. Information on experts involved in clinical, nonclinical studies. Environment risk assessment. Pharmacovigilance Certificate of pharmaceutical product (CoPP) Pricing Response to questions asked by SFDA (in any)	General information	Company profile Number of manufacturing sites owned by company Address of each site Legal and commercial relations with all sites Manufacturing license with date in country of origin (COO). Address of applicant with contact details. GMP certificate from country of origin. Number of employees in different section and their qualification details. Graphic design and flow of manufacturing line of site. List of all products manufactured at company’s site or contract manufacturing organization (if any) or other marketing authorization holder (MAH). Relationship with MAHs. Any previous inspection by GCC health authorities or Arab health authorities.	Information required	Reference standard with CoA. Finished product sample. Patient information leaflet (English and Arabic). Source of supply of API and inactive ingredients. Raw material specifications. Finished products’ specifications with quality control methods. Stability data: Long termthree batches (two pilot and one production). Accelerated studies: six months, same three batches, used for longterm studies. Bioequivalence study data.	Administrative information	Distributor of product in UAE. Manufacturing site. Marketing authorization holder and power of attorney. Manufacturer of API. Regulatory status. Price list. Declaration (In accordance with the medicines’ regulations of Drug Control Department- Ministry of Health -UAE)
Presentation	**a. Hard copy:** Bounded in a ring binder (A4 size, 2 ring binders). Not more than 300 pages in each binder.	Information to Ministry of Health authorities	MOH authorities should be informed about any sale, merge, takeover or any legal or commercial action concerning the company or its site within 90 days of the action.	Certificates	WHO-CoPP. CoA. Certificate of suitability for TSE free product. Legalized price list. Certificate of alcohol content. Composition certificate. List of countries where product has been registered.		
	**b. Soft copy:** CD-ROM/ DVD. Two soft copies and one hard copy. (For NCE, biologics and biosimilars- only module 1, 2 and 3 in hard copy). Media should be autostartable and not be bootable. Must be ‘virusfree’ and must not be passwordprotected. Language- English or Arabic.	Product description	Number of submitted files. Number of submitted samples. Table of content (index) of submitted files.			Product dossier	Drug product information. Packaging, patient information leaflet, labeling. Storage condition and shelf-life. Composition. Ingredients of animal origin. Leaflet information. Pharmacological properties. Bioequivalence details for generic product
Certificates	Authenticated by the Ministry of health of the country of origin and additionally by Saudi Arabian embassy. Following Certificates required: CoPP/ free sale certificate. CoA. Pork-free declaration. Price list.	Contents of file	WHO-certificate of pharmaceutical product (CoPP) issued by country of origin and legalized by any one GCC embassy. Summary of product characteristics (SmPC). Separate file for quality control laboratory including all quality data and information e.g. certificate of analysis, validation report etc. Full description about API, and excipients excipients. Description about vehicles and carriers used. Method of manufacturing. Name and address of involved CROs (if any). Description of outer pack and accessories used. Concentration of product (per unit mass or volume). Source of starting material. Proposed label. Product patient leaflet (Arabic/English). Toxicological data (for NCE and newly introduced generic drugs only). Bioequivalence study reports (for generic drug registration). Animal source (if any). Price certificate authenticated by health authorities of country of origin.				

**Table 6:** Enclosures required for drug registration in five Gulf Cooperation Council countries

Sr. no.	Enclosures	Saudi Arabia	Kuwait	Uae	Oman	Bahrain
1	Control specification and method of analysis	R	R	R	R	NR
2	Certificate of analysis attested by health authority and country of origin (CoO)	R	NR	R	NR	NR
3	Legalized free sale certificate issued by health authorities of CoO, indicating that product is registered and marketed with same name and composition.	R	R	R	R	R
4	Legalized certificate indicating that diluents used are allowed to be used in CoO	R	NR	NR	NR	R
5	Legalized price certificate issued by competent authority of CoO and attested by embassy including ex-factory price, wholesale price in CoO	R	R	R	NR	R
6	Retail/ public price in CoO	R	NR	R	NR	R
7	Export price to country and neighboring countries	R	NR	R	NR	R
8	Stability studies in various defined conditions	R	R	R	R	R
9	Storage conditions	R	R	R	R	R
10	Name of developed countries in which the product is registered	R	R	R	R	R
11	Abstract from scientific references about product	R	R	R	R	R
12	Sealed sample of product and copies of label	R	R	R	R	R
13	Quantity specified for each pack and outer pack of product	R	R	NR	NR	R
14	Leaflet in Arabic and English, including;					
	A. Name of product	R	R	R	R	R
	B. Composition	R	R	R	R	R
	C. Mode of action	R	R	R	R	R
	D. Effect	R	R	NR	R	R
	E. Indications	R	R	NR	NR	R
	F. Contraindications	R	R	NR	R	R
	G. Precautions	R	R	NR	R	R
	H. ADR	R	R	NR	R	R
	I. Antidote	R	R	NR	NR	R
	J. Dosage and administration	R	NR	NR	R	R
15	Product labeling	R	R	R	R	R
16	Bioavailability studies	R	R	R	R	R
17	Scientific basis of justifying the formulation of combination product	R	R	R	NR	NR
18	Post marketing surveillance	R	NR	NR	NR	NR
19	Certificate of sterility and pyrogen free pharmaceutical product	NR	R	R	NR	NR
20	Copy of reference pharmacopoeia	R	R	R	NR	NR
21	CTD format for dossier submission	R[9]	NR	NR	NR	NR
22	eCTD or NeeS (non eCTD electronic submission)	R[9]	NR	NR	NR	NR

R: Recommended by regulatory authority, NR: Generally, not recommended by regulatory authority

## CONCLUSION

The GCC market is very lucrative in terms of benefits offered to the Indian pharmaceutical industry. With large pharmaceutical markets like the US, Europe and Japan getting saturated, it is the need of the hour that the Indian pharmaceutical industry in general and pharmaceutical companies in particular tap the opportunity of this “emerging” GCC market.
